# Effect of Laser and Conventional Office Bleaching and Polishing on the Color Change of Stained Nanohybrid and Microhybrid Composite Resin

**DOI:** 10.1155/2023/9912560

**Published:** 2023-07-28

**Authors:** Shakiba Farahani, Taraneh Faghihi, Ladan Ranjbar Omrani, Nasim Chiniforush, Elham Ahmadi, Mandana Karimi, Mahdi Abbasi

**Affiliations:** ^1^Department of Restorative Dentistry, Dental School, Tehran University of Medical Sciences, Tehran, Iran; ^2^Department of Pediatric Dentistry, Faculty of Dentistry, Ardabil University of Medical Sciences, Ardabil, Iran; ^3^Department of Surgical Sciences and Integrated Diagnostics, University of Genoa, Genoa, Italy

## Abstract

**Aim:**

The present study investigated the effects of laser and conventional in-office bleaching, and polishing on the color of stained composite resin.

**Materials and Methods:**

A microhybrid composite (Clearfil AP-X) and a nanohybrid composite (Grandio) were selected. Twenty-four discs (2 × 10 mm) for each composite were prepared. The samples were immersed in coffee solution (25 g of coffee in 250 mL water) for seven days. Then the samples were divided into three groups (*n* = 8) and the stains were removed using bleaching (with Opalescence Xtra Boost), diode laser irradiation with Heydent material and a Sof-Lex polishing kit. The *L* ^*∗*^*a* ^*∗*^*b* ^*∗*^ color parameters were determined using a spectrophotometer before and after immersion and after stain removal procedures, and the overall color changes (*ΔE*) were calculated. The data were analyzed with two-way analysis of variance.

**Results:**

In the Clearfil composite resin group, the mean *ΔE* compared to the baseline using in-office bleaching, laser irradiation, and Sof-Lex polishing kit were 3.31, 3.35, and 4.93, respectively. These values with the Grandio composite resin were 3.31, 6.35, and 4.57, respectively. The highest capacity to remove stains was related to the conventional in-office bleaching method. Grandio composite resin underwent more color changes than Clearfil composite resin significantly (*P*-value < 0.05).

**Conclusion:**

Both composite resins exhibited color changes after immersion in the discoloring solution. However, after staining-removing procedures, the *ΔE* values decreased. Decreases in the *ΔE* values were not sufficient to restore the color to that before immersion in the discoloring solution with any stain-removing methods.

## 1. Introduction

Composite resins are the most commonly used restorative materials due to their good aesthetic appearance, adequate strength, conservative nature, and moderate cost [1]. However, color changes of composite resin restorations in the long term lead to patient dissatisfaction and are one of the most common reasons for replacing or reconstructing restorations, which wastes time and imposes extra costs on patients [2]. The success of composite resin materials depends significantly on their color stability over time [3].

Composite resins undergo external or internal color changes. It has been demonstrated that intrinsic color changes in restorative materials are related to their resin content and water sorption. Extrinsic color changes, also called staining, occur due to the superficial and deep absorption of water-soluble discoloring agents after contact with solutions containing pigments, such as tea, coffee, and some alcoholic drinks [4]. Another important factor that causes external color change is smoking. Smoking significantly affects color stability and resin-based composites are subjected to irreversible color change if exposed to smoke [5].

The resin matrix structure and the properties of the fillers, including their size and type, play a role in the stainability of composite resins, which is also affected by the pH of solutions and alcohol compounds [6]. In addition, stainability of the resin matrix is related to their degree of conversion and chemical properties, which affect the water sorption rate [7].

Some methods suggested to overcome color change problems in composite resins include replacing the restoration, repolishing, and bleaching procedures. Bleaching is one of the most effective and relatively conservative procedures in dentistry [8, 9]. The bleaching agent usually contains peroxide (for example, hydrogen peroxide, carbamide peroxide, and sodium perborate). The procedure is carried out using in-office or at-home techniques. In-office bleaching is carried out with 35%‒38% hydrogen peroxide placed on the tooth surface for 30‒45 min, and the bleaching agent might be light- or laser-activated [10]. Hydrogen peroxide is a strong antioxidant, which is disintegrated into free radicals that attack organic molecules; and it also releases other radicals. These radicals convent the large pigmented molecules responsible for color changes in composite resins to small pigmented molecules through an oxidative process and reduction reactions [11].

Some studies have shown that the effect of bleaching agents containing peroxide on the color of tooth-colored materials is not noticeable [12, 13]. However, some other studies have reported significant effects of these agents on composite resin materials. Differences in these effects have been attributed to the volume of the resin matrix and the filler type [14–16].

In order to enhance bleaching procedure, various methods are used. One of these methods is photochemistry by lasers of various wavelengths. As far as the bleaching process is concerned, the absorption of laser light in the bleaching gel is needed [17]. Hydrogen peroxide is optically transparent; therefore, without adding a coloring agent, one cannot expect it to absorb visible or near-infrared laser light to any great extent. By choosing appropriate chromophores, a range of processes can be triggered [18]. Argon, KTP (potassium titanyl phosphate), and diode lasers are most commonly used for this purpose. Diode lasers have a monochromatic characteristic which reduce the risk of pulpal damage due to overheating [19]. Different powers and wavelengths of diode lasers are used for laser bleaching. It has been shown that using a low-intensity red diode laser (660 nm) with a green bleach gel resulted in a significant change of color (*ΔE* was increased from 5.4 to 7.2 after 1 week) [20].

Due to the increasing consumption of colored beverages, finding a less invasive way to replace stained composite restorations is essential. According to the review of the literatures, no study has been conducted to investigate the effect of laser bleaching in this regard compared to previous methods such as conventional bleaching and polishing. As a result, this experimental study was designed to evaluate and compare the effects of laser and conventional in-office bleaching, and polishing on the color of stained composite resin.

## 2. Materials and Methods

By using the two-level factorial design option to determine the sample size in Minitab software, considering *α* = 0.05, *β* = 0.05, effect size = 1/3, and average standard deviation equal to one; the minimum sample volume required for each of the subgroups was estimated to eight [21].

In the present in vitro study, a silicon mold was used to prepare 24 disc-shaped samples measuring 10 mm in diameter and 2 mm in depth from a nanohybrid (Grandio, Voco GmbH, Cuxhaven, Germany) and a microhybrid (Clearfil AP-X, Kuraray Noritake Dental, Tokyo, Japan) composite resin in A2 shade. The composition of these two composite resins are shown in Table 1. The molds were placed on transparent celluloid Mylar matrix bands and a glass slab and packed with composite resin using a plugger (NB mini plugger, Bisco, USA) in one layer, slightly higher than the mold margin. The filled mold was covered with another matrix band and glass slab (200 g) and mildly pressed by finger pressure for 20 s to extrude extra material from the mold. The disc samples were light-cured using a light-emitting diode unit (Woodpecker LED Curing, Guilin Woodpecker Medical Instrument Co., Guilin, China) for 20 s from the upper and lower surfaces at a light intensity of 1,000–1,700 mW/cm^2^ at 385‒515 nm wavelength from a distance of 1 mm from the sample's surface to complete the polymerization process. The light-curing unit's output was tested with a radiometer (LM1, Woodpecker, China). Excess composite resin was removed with a scalpel blade. The samples were polished with coarse, medium, fine, and superfine aluminum oxide discs (Sof-Lex, 3M ESPE, USA) in a low-speed handpiece with interrupted movements. Each disc was used 12 times on each surface. Then the samples were immersed in distilled water at 37°C for 24 hr.

Then the samples of each composite resin were randomly assigned to three groups (*n* = 8).

The samples of all groups were immersed in 20 mL coffee solution (Nescafe Classic Nestle), in capped containers at 37°C for 7 days in a dark environment. The solutions were changed every day. To prepare the coffee solution, 25 g of coffee was dissolved in 250 mL of boiling water and filtered for 10 min before pouring it into the container. After immersion, the samples were rinsed under running water with a powered toothbrush (PRO3 3500, Oral-B, Germany) for one minute and immersed in distilled water at 37°C for 24 hr. Then each group underwent different stain removal method:

  Group 1 (in-office bleaching): bleaching was done with Opalescence Xtra Boost (Ultradent Products Inc., USA) containing 40% hydrogen peroxide three times, each time for 20 min, according to the manufacturer's instructions. After removing the bleaching agent, the samples were rinsed in distilled water for 30 s to remove the bleaching agent completely.
  Group 2 (laser activated bleaching): the laser-activated JW power bleaching gel (Heydent GmbH, Germany) containing 35% hydrogen peroxide was applied in a layer 1.5–2-mm-thick on the sample surface. The diode laser (Cheese TM, Wuhan Gigaa Optronics Technology Co, Ltd, China) was applied with a wavelength of 810 nm and output power of 1.5 W in continues mode at a distance of 6 mm. The irradiation was done for 30 s, followed by a rest interval of 1 min. This process was done three times for each sample. After that, bleaching gel remained on the surface for 7 min. After removing the bleaching agent, the samples were rinsed in distilled water for 30 s to remove the bleaching agent completely.
  Group 3 (polishing with a Sof-Lex kit): the samples were polished with coarse, medium, fine, and superfine aluminum oxide discs (Sof-Lex, 3M ESPE, USA), as described above. Finally, the samples were rinsed with distilled water for 30 s.

The color of each specimen was measured at baseline (prior to staining, *T*_0_), after staining (*T*_1_), and after stain-removal procedures (*T*_2_). A dental spectrophotometer (VITA Easyshade LITE, Zahnfabrik H. Rauter GmbH & Co., Germany) was used to record the CIE *L*^*∗*^*a*^*∗*^*b*^*∗*^ coordinates of all samples. Color measurements were performed under D65/2° viewing conditions with a white background. The spectrophotometer tip was placed at 90° on the center of each sample. Calibration was carried out by placing the probe tip on the calibration port of the instrument (one standard for calibration) before carrying out measurements for each sample. The color changes of each sample were measured three times and the mean of the three measurements was reported as the final data.

The overall color change between *T*_0_ and *T*_1_ (*ΔE*_1_), *T*_1_ and *T*_2_ (*ΔE*_2_) and *T*_0_ and *T*_2_ (*ΔE*_3_) was calculated using the following formula:

(1) $$\begin{array}{c} \Delta E={\left[{\left(\Delta L^{*}\right)}^{2}+{\left(\Delta a^{*}\right)}^{2}+{\left(\Delta b^{*}\right)}^{2}\right]}^{1/2}. \end{array}$$

*L* ^*∗*^ indicating the lightness parameter, *a* ^*∗*^ indicating the red–green parameter, and *b*^*∗*^ indicating the yellow–blue parameter.

Data were statistically analyzed using two-way analysis of variance (ANOVA) to assess the effect of the type of composite resin and the stain removal method. *P* < 0.05 was considered statistically significant. The clinically acceptable color change was defined at *ΔE* = 3.3 [22].

## 3. Results

**Figure 1:**
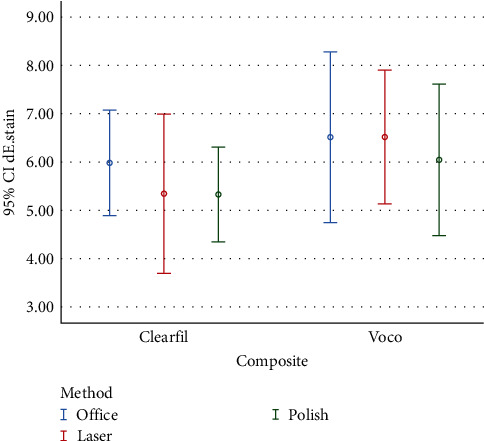
Graph of mean color change of two composite resins after immersion in coffee solution.

Color change of composite resins after immersion in coffee solution compared with baseline (*ΔE*_1_) are presented in Figure 1 and Table 2.

According to the results of two-way ANOVA, the effects of composite resin type on *ΔE* was not significant (*P* = 0.11) after immersion in the coffee solution compared to the baseline (Table 3).

**Figure 2:**
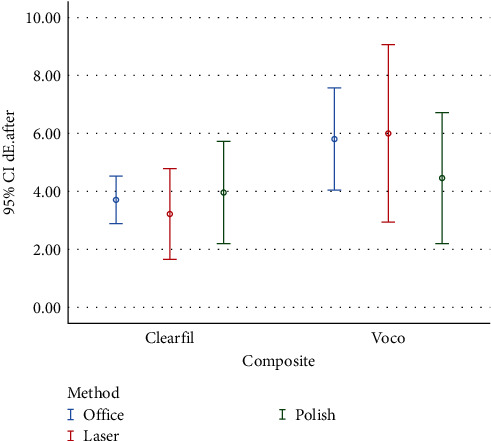
Graph of mean color change after stain-removal procedures compared with after staining.

Color change of composite resins after stain removal compared with after immersion in coffee solution (*ΔE*_2_) are presented in Figure 2 and Table 4.

After stain removal, the effect of composite resin type on *ΔE* was significant (*P* < 0.01), while the effects of the stain-removal method (*P* = 0.78) and the cumulative effects of composite resin type and stain-removal method (*P* = 0.36) were not significant at this stage (Table 5).

**Figure 3:**
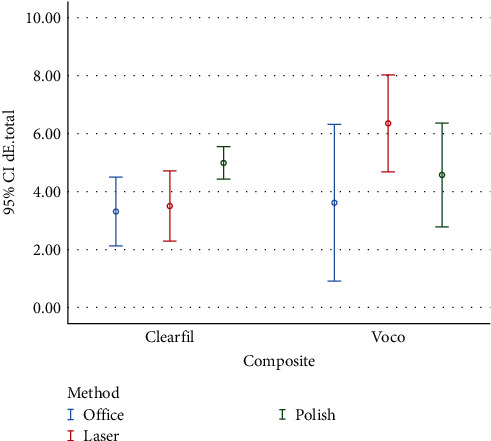
Graph of mean color change after stain-removal procedures compared with baseline.

Color change of composite resins after stain removal compared with baseline (*ΔE*_3_) are presented in Figure 3 and Table 6.

Evaluation of the overall color changes after stain removal compared to baseline showed the effect of composite resin type was significant (*P* < 0.05). However, the effect of the stain-removal method (*P* = 0.08) and the cumulative effect of composite resin type and stain-removal method (*P* = 0.09) were not significant (Table 7).

## 4. Discussion

The present study investigated the color changes of one microhybrid and one nanohybrid composite resin using three stain-removal methods to correct their color. For this purpose, the CIEL ^*∗*^*a* ^*∗*^*b* ^*∗*^ system was used to evaluate color changes. In this system, color is reported using three parameters: *L ^*∗*^* indicates “value” from white to black; *a* ^*∗*^ indicates the green–red parameter; and *b* ^*∗*^ indicates the blue–yellow parameter. The overall color changes (*ΔE*) are determined using the above parameters. *ΔE* indicates relative color changes that an observer can perceive and report after immersing a material in different solutions or at different time intervals. Therefore, *ΔE* is more accurate than calculating each of the *L* ^*∗*^*a* ^*∗*^*b* ^*∗*^ parameters [23]. Different studies have reported different values for perceiving color changes by an observer with normal vision. However, the clinically acceptable level of color changes has been reported at *ΔE* ≤ 3.3 [22]. Color perception is a physiological issue, and there are significant differences between individuals and between different times in one individual. However, evaluating color parameters in vitro with CIEL ^*∗*^*a* ^*∗*^*b* ^*∗*^ system has the advantage of eliminating mental errors during color change evaluations [22, 23].

According to the results of this study, color changes after immersion in coffee soloution were not significantly different between Grandio and Clearfil composite resins; however, after removing the stains by the in-office polishing–bleaching and laser bleaching, color changes were greater compared to the baseline values and also significantly higher in Grandio composite resin. This might be attributed to the deeper absorption of pigments or greater intrinsic discoloration in Grandio composite resin.

Recent studies showed that the penetration of water and pigments was higher in nanohybrid composite resin than in microhybrid composite resin, which was attributed to a lack of complete silanization of nanoparticle clusters and prepolymerized fillers in nanohybrid composite resin. These properties are also seen in Grandio composite resin [24–26]. In addition, some researchers have reported that an increase in the size of filler particles increases the composite resin's color stability by decreasing the ratio of the organic filler matrix [26, 27].

In another studies there was no significant difference between microhybrid and nanohybrid composite resins' color stability after staining and bleaching. Such a discrepancy between the results might be attributed to the protocol of the study like different soloutions, bleaching gels, hydrogen peroxide concentration and so forth [7, 12, 28].

The mechanism of color changes in restorative materials after applying bleaching agents has not been completely elucidated. Free peroxyl radicals might cause oxidative cleavage in polymer chains, and the free radicals finally produce water and oxygen, accelerating the hydrolytic degradation of composite resins. Therefore, composite resins with a higher resin content become susceptible to degradation and, as a result, to color changes. For example, composite resins with a higher filler content undergo more color changes in the long term [29, 30].

The coffee soloution was used in this study as it causes higher color changes compared to other common beverages in long time [31]. Coffee has yellow pigments with a high polarity that are released with a delay and are compatible with the polymer structure and can penetrate it. Therefore, color changes due to coffee occur due to surface and deep absorption [25].

In this study, the samples were immersed in the coffee solution for one week, and the solution was changed daily. However, such a protocol cannot be implemented clinically because, under clinical conditions, composite resin restorations are exposed to discoloring solutions periodically. In addition, the saliva and other solutions dilute these solutions in the oral cavity. Although the present study collected important data on the effects of laser, Sof-Lex discs, and bleaching agents on removing stains from composite resin surfaces. The discoloring solution in the present study (coffee) does not represent all the solutions the teeth might be exposed to clinically [24].

According to the results of this study, laser irradiation to remove stains could not restore the color of the composite resin samples to that before immersion in the discoloring solution. Laser irradiation in bleaching procedures accelerates the procedure. Although bleaching can remove external stains from composite resin surfaces, its ability to effectively bleach composite resin materials is limited [21, 32]. Bleaching agents should be cautiously used to remove stains from the surfaces of composite resin restorations because they might increase surface roughness under these conditions and the susceptibility of composite resin surfaces to absorb pigments resulting from foodstuff and drinks and other pigment sources [21]. However, Peng et al. [33] reported that using a light source increased the bleaching efficacy; therefore, it could decrease the color changes of stored composite rein samples to levels that could not be clinically perceived, which was not confirmed in the present study.

The mechanism of removing stains by bleaching agents and Sof-Lex discs is different. Sof-Lex discs and pumice remove stains through surface abrasion. Sof-Lex abrasive agents include aluminum oxide particles. The Sof-Lex system abrasive agents should be harder than the composite resin fillers to effectively remove stains from the surfaces of composite resin materials; otherwise, the polishing agent will only remove the soft resin matrix, and the filler particles will remain protruded on the composite resin surface. In a study by Al-Nahedh and Awliya [21], similar to the present study, Sof-Lex discs could remove stains from the surface of nanofilled Filtek Supreme composite resin, which was attributed to the degradation of organic fillers and initial nanofillers during the polishing process and the successful removal of stains from the superficial layer of composite resin.

In the present study, after polishing with Sof-Lex discs, in-office bleaching, and laser bleaching, the color of composite resin samples improved, but it was not able to revert to the original color before staining. In addition, there were no significant differences in the efficacy of the three stain-removal methods.

In previous studies, polishing has been as effective as bleaching or better than that in improving the color of composite resins [21, 34–36], consistent with the present study. However, in another study in-office bleaching was found to be more effective than repolishing in the restitution of the color [37]. Also, in one study, bleaching technique returned composite color close to baseline, but polishing permitted residual stain to remain. It could be related to different mehods. In the mentioned study, pumice was used for polishing instead of Sof-Lex discs [38].

On the other hand, all the samples used in the present study were flat, while composite resin restorations in clinical conditions have irregular shapes, and their surfaces might be convex or concave. Also, polishing procedures are very easy in vitro, but difficult in the clinic. In addition to these factors, thermal cycles, wear, polishing method, and abrasion affect color changes in composite resins. However, sufficient studies have not evaluated the effects of different polishing methods on surface roughness and their relationship with color changes in composite resins [37, 39, 40].

It should be noted that the bleaching process improved the color of the composite restorations but it might cause degradation and microcracks in composite resin and, finally, exert detrimental effects on the composite resin's acceptability clinically, which should be investigate in the future studies [41, 42]. Furthermore, in future studies, color stability should also be investigated with materials layered on a substrate thus reproducing their clinical behavior [43].

## 5. Conclusion

Both composite resins exhibited color changes after immersion in the discoloring solution. After stain-removal procedures from these surfaces, the color changes decreased. However, the color changes in none of the methods were adequate to restore the initial colors of the samples before immersion in the discoloring solution.

## Figures and Tables

**Table 1:** Charachteristics of composite resins used in the study.

Material (code)	Type	Organic matrix	Fillers	Filler amount (wt%)	Manufacturer
Grandio (GRA)	Nanohybrid	Bis-GMA, Bis-EMA, and TEGDMA	Glass ceramic, silicon dioxide nanoparticles	87	Voco GmbH, Cuxhaven, Germany
Clearfil AP-X (AP-X)	Microhybrid	Bis-GMA and TEGDMA	Barium glass, silica, colloidal silica	85.5	Kuraray Noritake Dental, Tokyo, Japan

**Table 2:** Mean color change of two composite resins after immersion in coffee solution (*ΔE*_1_).

Composite resin	Method	Mean ± SD	Maximum	Minimum
Clearfil	Polishing	5.33 ± 1.17	7.04	3.49
	Office bleaching	5.98 ± 1.3	7.97	4.03
	Laser bleaching	5.35 ± 1.97	8.38	3.3

Grandio	Polishing	6.05 ± 1.88	8.32	2.82
	Office bleaching	6.51 ± 2.11	9.4	3.73
	Laser bleaching	6.52 ± 1.66	8.47	4.46

**Table 3:** Two way ANOVA test of mean color change of two composite resins after immersion in coffee solution (*ΔE*_1_).

Source	Type III sum of squares	*df*	Mean square	*F*	Sig.
Corrected model	11.223^a^	5	2.245	0.761	0.583
Intercept	1,702.706	1	1,702.706	577.144	0.000
Composite	7.813	1	7.813	2.648	0.111
Method	2.538	2	1.269	0.430	0.653
Composite ^*∗*^ method	0.871	2	0.435	0.148	0.863
Error	123.909	42	2.950		
Total	1,837.839	48			
Corrected total	135.132	47			

^a^ *R* ^2^ = 0.083 (adjusted *R*^2^ = −0.026).  ^*∗*^Interacion between composite and method and its effect on *ΔE*. ANOVA, analysis of variance.

**Table 4:** Mean color change after stain-removal procedures compared with after staining (*ΔE*_2_).

Composite resin	Method	Mean ± SD	Maximum	Minimum
Clearfil	Polishing	3.96 ± 2.11	7.06	1.18
	Office bleaching	3.71 ± 0.98	5.68	2.76
	Laser bleaching	3.22 ± 1.87	6.5	0.92

Grandio	Polishing	4.46 ± 2.7	8.85	1.5
	Office bleaching	5.81 ± 2.11	9.12	3.56
	Laser bleaching	6.0 ± 3.31	10.99	1.79

**Table 5:** Two way ANOVA test of mean color change after stain-removal procedures compared with after staining (*ΔE*_2_).

Source	Type III sum of squares	*df*	Mean square	*F*	Sig.
Corrected model	49.826^a^	5	9.965	1.941	0.108
Intercept	960.806	1	960.806	187.170	0.000
Composite	37.647	1	37.647	7.334	0.010
Method	2.550	2	1.275	0.248	0.781
Composite ^*∗*^ method	10.678	2	5.339	1.040	0.363
Error	210.467	41	5.133		
Total	1,210.185	47			
Corrected total	260.293	46			

^a^ *R* ^2^ = 0.191 (adjusted *R*^2^ = 0.093).  ^*∗*^Interacion between composite and method and its effect on *ΔE*. ANOVA, analysis of variance.

**Table 6:** Mean color change after stain-removal procedures compared with baseline (*ΔE*_3_).

Composite resin	Method	Mean ± SD	Maximum	Minimum
Clearfil	Polishing	4.93 ± 0.61	5.63	4.22
	Office bleaching	3.31 ± 1.42	5.34	2.02
	Laser bleaching	3.35 ± 1.25	4.14	1.44

Grandio	Polishing	4.57 ± 2.14	8.6	1.71
	Office bleaching	3.31 ± 2.41	7.03	3.03
	Laser bleaching	6.35 ± 1.81	8.5	3.14

**Table 7:** ANOVA test of mean color change after stain-removal procedures compared with baseline (*ΔE*_3_).

Source	Type III sum of squares	*df*	Mean Square	*F*	Sig.
Corrected model	50.442^a^	5	10.088	2.639	0.037
Intercept	903.258	1	903.258	236.266	0.000
Composite	9.769	1	9.769	2.555	0.046
Method	20.480	2	10.240	2.678	0.081
Composite ^*∗*^ method	22.555	2	11.277	2.950	0.094
Error	156.745	41	3.823		
Total	1,095.540	47			
Corrected total	207.188	46			

^a^ *R* ^2^ = 0.243 (adjusted *R*^2^ = 0.151).  ^*∗*^Interacion between composite and method and its effect on *ΔE*. ANOVA, analysis of variance.

## Data Availability

Data supporting this research article are available from the corresponding author on reasonable request.
